# The potential impact of the Affordable Care Act and Medicaid expansion on reducing colorectal cancer screening disparities in African American males

**DOI:** 10.1371/journal.pone.0226942

**Published:** 2020-01-24

**Authors:** Wizdom Powell, Leah Frerichs, Rachel Townsley, Maria Mayorga, Jennifer Richmond, Giselle Corbie-Smith, Stephanie Wheeler, Kristen Hassmiller Lich

**Affiliations:** 1 Health Disparities Institute and Department of Psychiatry, University of Connecticut, Farmington, CT, United States of America; 2 Department of Health Policy and Management, Gillings School of Global Public Health, University of North Carolina at Chapel Hill, Chapel Hill, NC, United States of America; 3 Department of Industrial and Systems Engineering, North Carolina State University, Raleigh, NC, United States of America; 4 Department of Health Behavior, Gillings School of Global Public Health, University of North Carolina at Chapel Hill, Chapel Hill, NC, United States of America; 5 Center for Health Equity Research, University of North Carolina at Chapel Hill, Chapel Hill, NC, United States of America; 6 Department of Medicine, University of North Carolina at Chapel Hill, Chapel Hill, NC, United States of America; 7 Department of Social Medicine, University of North Carolina at Chapel Hill, Chapel Hill, NC, United States of America; Brown University, UNITED STATES

## Abstract

Few investigations have explored the potential impact of the Affordable Care Act on health disparity outcomes in states that chose to forgo Medicaid expansion. Filling this evidence gap is pressing as Congress grapples with controversial healthcare legislation that could phase out Medicaid expansion. Colorectal cancer (CRC) is a commonly diagnosed, preventable cancer in the US that disproportionately burdens African American men and has substantial potential to be impacted by improved healthcare insurance coverage. Our objective was to estimate the impact of the Affordable Care Act (increasing insurance through health exchanges alone or with Medicaid expansion) on colorectal cancer outcomes and economic costs among African American and White males in North Carolina (NC), a state that did not expand Medicaid. We used an individual-based simulation model to estimate the impact of ACA (increasing insurance through health exchanges alone or with Medicaid expansion) on three CRC outcomes (screening, stage-specific incidence, and deaths) and economic costs among African American and White males in NC who were age-eligible for screening (between ages 50 and 75) during the study period, years of 2013–2023. Health exchanges and Medicaid expansion improved simulated CRC outcomes overall, though the impact was more substantial among AAs. Relative to health exchanges alone, Medicaid expansion would prevent between 7.1 to 25.5 CRC cases and 4.1 to 16.4 per 100,000 CRC cases among AA and White males, respectively. Our findings suggest policies that expanding affordable, quality healthcare coverage could have a demonstrable, cost-saving impact while reducing cancer disparities.

## Introduction

Our nation is embattled over the future of its healthcare system. Public concern has been mounting about the potential impacts of an ACA repeal on populations at risk for health and healthcare disparities. In addition to expanding private healthcare coverage, ACA legislation afforded states a historic opportunity to expand Medicaid to childless adults with incomes at or below 138 percent of the federal poverty level (FPL) [1]. Thus far, thirty-seven states and the District of Columbia have expanded Medicaid eligibility [2]. Expanded eligibility is having a disproportionately positive impact on adult men compared to women because men have historically been less often Medicaid-eligible [1]. Indeed, Medicaid expansion states are experiencing post-ACA gains in insurance coverage among newly eligible childless men [3]. For example, in the setting of expanded Medicaid eligibility, the probability of insurance coverage among men increased by 14.2 percentage points [3]. Furthermore, coverage gains are most pronounced among racial/ethnic minority men (e.g., non-Hispanic Blacks or African Americans)—populations with well-documented racial disparities in conditions amenable to treatment upon early detection [4, 5].

Findings from a notable quasi-experimental study suggested that the most significant gains in health insurance coverage are attributable to full ACA implementation with Medicaid expansion as opposed to ACA implementation alone [6]. Although extant studies explore Medicaid expansion’s impacts on healthcare access, utilization, affordability, and economic outcomes [7], they have largely failed to fully illuminate the effects of this policy intervention on specific health outcomes where persistent disparities have been documented. Moreover, to our knowledge, few investigations explore the potential impacts of ACA policy scenarios on health outcomes in states that chose to forgo Medicaid expansion. Filling this evidence gap is pressing as Congress grapples with controversial healthcare legislation that could phase out Medicaid expansion. Analyzing and quantifying the implications of opting in or out of health policy alternatives could richly inform healthcare reform debates.

Analysis of policy alternatives can be challenging with conventional statistical and econometric methods because they require observed data, yet our current datasets fail to provide the full scope of measures required, do not allow us to estimate unobserved scenarios (e.g., North Carolina did not expand Medicaid), and are unable to capture downstream impacts on cancer incidence and mortality. In contrast, significant insights about the potential impacts of ACA and Medicaid expansion on health disparities can be gleaned from individual-based simulation. Individual-based simulation models estimate population-level outcomes over time using information about a population’s demographic structure and behaviors, which are shaped by context. The models provide flexibility for integration of diverse sources of data and estimate plausible projections of future health outcomes. Many studies have demonstrated the utility of individual-based simulation models for exploring population-level impacts of policy interventions [8]. Individual-based simulation models were used prior to ACA to estimate potential impacts on enrollment and insurances rates [9–11]. However, few studies have used the methods to estimate impacts of ACA on preventable health outcomes such as colorectal cancer (CRC) [12, 13]. CRC is an ideal condition for which to study using simulation because investments in screening can have long-term implications on CRC incidence and mortality outcomes that are not captured by only a few years of short-term observational data. Utilizing these methods, our investigation addresses critical evidentiary gaps by exploring the potential impact of ACA’s health exchanges and Medicaid expansion on improving CRC screening and reducing disparities in CRC incidence and death among African American men.

We use CRC as a test case because it is one of the most commonly diagnosed, preventable cancers in the US [14], representing about 8.0% of new cancer diagnoses in the country [15]. Compared to other race, ethnic, and gender groups, African American men have the highest CRC incidence and mortality rates, which are 27% and 52% higher, respectively than non-Hispanic White men [14, 16]. Using CRC as a test case is supported by evidence indicating that recently observed reductions in CRC incidence among African American men and other population groups are attributable to screening [17]. Also, as a result of the ACA and overwhelming evidence of CRC screening effectiveness, health plans started after September 23, 2010 are required to cover billable CRC screening costs among age-eligible individuals [18, 19]. Although states have some discretion in determining screening type and timing, Medicaid programs now also cover CRC screening [19].

Expansion states are experiencing an uptick in CRC screening (+4.3 percentage points) among adults ages 50–64 [20]. Thus, non-expansion states may be missing a critical opportunity to reduce CRC mortality, especially in populations with the most persistent disparities in CRC screening. We use North Carolina (NC), one of the non-expansion states, as a case example to estimate the potential for ACA health exchanges and Medicaid expansion to reduce CRC deaths, incidence, and screening disparities.

## Materials and methods

### Study design

We used an individual-based simulation model to estimate CRC screening, stage-specific incidence, and deaths among African American and White males in NC. A more comprehensive overview of the simulation model is published elsewhere [21]. Briefly, the natural history portion of the simulation model was estimated and calibrated using adenoma prevalence data from autopsy studies and CRC incidence data in years prior to initiation of widespread screening [22, 23]. The model simulates the life course of each individual and tracks polyp and adenoma progression, of which some may eventually become malignant. Preclinical cancer can progress through stages I to IV, and CRC can be detected by either symptoms or CRC screening at any stage. For the current study, we incorporated recent evidence differentiating polyp incidence rates by race [24].

Using a combination of health insurance claims and Behavioral Risk Factor Surveillance System (BRFSS) data, we differentiate factors affecting probability of individual receipt of alternative CRC screening modalities (fecal testing or colonoscopy) and the predicted probability of compliance (i.e., whether or not CRC screening is completed) into the simulation model based on individual sociodemographic factors of race, gender, and insurance type as well as county-level factors affecting practice patterns and distance to endoscopy centers [25, 26]. For each individual, it was randomly determined whether they obtained CRC screening based on their predicted probability at intervals appropriate for their preferred modality (e.g., each year for fecal testing and every 10 years for colonoscopy). The decennial probability of screening was implemented as two 5-year opportunities for screening in order to better represent actual screening patterns observed in claims data (i.e., individuals did not screen at exactly 10-year intervals). Age of first screening with colonoscopy was modeled with a probability based on claims data analysis that showed it is well-distributed between 45 and 55. Compliance with diagnostic testing and surveillance following CRC screening with abnormal or positive results were set at 50% and 80% respectively, in accordance to findings in recent studies [27–29].

### Study population

We used the RTI Synthetic Household Population, which provides an accurate sociodemographic representation of households and persons (e.g., household income, household size, gender, race) throughout the US [30]. For our analysis, we focused on White and African American males in NC who were age-eligible for screening (between ages 50 and 75) during the study period, years of 2013–2023. For simplification, we assumed that county of residence, income, and marital status were static. Each synthetic individual was probabilistically assigned to one of six distinct insurance types based on age, household income and size, and insurance status based on additional census data [21]: (1) privately insured, (2) Medicaid, (3) Medicare, (4) dually insured through Medicare and Medicaid, (5) low income uninsured, (6) other uninsured. As each individual turned 65 years of age, they either became insured with Medicare or dually insured through Medicare and Medicaid, dependent on their prior insurance type and income level as related to Medicaid eligibility.

### Model calibration

We compared the claims-based simulation model’s predicted probability of being up-to-date with CRC screening to the percent of NC residents aged 50–75 years who reported being up-to-date with CRC screening in biannual data from the BRFSS survey from 2002 to 2012 [21]. The BRFSS data was adjusted to account for over-reporting due to bias in self-reported preventive service use measures [21]. The simulation model, which estimated screening compliance probabilities using private and public insurance claims data that are known to under-report screening, predicted the percent up-to-date with CRC screening as low, compared to the adjusted BRFSS data. Thus, we calibrated the simulation model by adding year-specific constants that increased each insured individual’s compliance probability to more closely approximate the adjusted percent up-to-date reported by BRFSS. We compared the CRC incidence from the model with historical cancer incidence data for NC. The model underestimated CRC incidence and thus provides conservative estimates of impacts.

### Control scenario

We modeled a control scenario to provide a baseline in the absence of ACA policy changes (i.e., prior to the existence of health exchange marketplaces and Medicaid expansion). The BRFSS data indicated that from 2002 to 2014, the percent up-to-date initially increased and then leveled off around 2012. Thus, for the control scenario (e.g., no ACA), we assumed CRC screening compliance probabilities remained stable after 2012.

#### ACA policy scenarios

We simulated five ACA policy scenarios. We simulated one scenario to evaluate the potential impact of increased insurance coverage through the implementation of the ACA’s health exchange insurance marketplaces only, beginning in 2014. We simulated four policy scenarios to evaluate the potential impact of Medicaid expansion if NC had expanded in 2014.

#### ACA health exchange scenario

To model the impact of the health insurance marketplaces, we used NC BRFSS data to determine the probability of an individual becoming newly insured in 2014 based on sex, age, income, race, and marital status, accounting for secular trends. To implement insurance change in the simulation model, we assigned a probability of obtaining healthcare coverage in 2014 to individuals who were uninsured prior to 2014 that matched the probabilities observed in BRFSS data. We assumed that increases of health insurance coverage in 2014 were due to both the newly available health insurance marketplace enrollments and an increase in public awareness about Medicaid coverage (i.e., the “woodwork effect”) [31]. Thus, in the simulation model, if an individual had a poverty level that qualified them for Medicaid and was identified as becoming newly insured in 2014 (based on their assigned probability), they were assumed to become insured through Medicaid. All other individuals were assumed to become newly insured though private insurance. In the absence of data to suggest otherwise, individuals who became newly insured were assumed to follow the CRC screening compliance and modality probabilities of their new insurance type, which were based on multilevel, multivariable statistical models as described above [25, 26, 32]. In our continued effort to simplify simulation of insurance coverage overall (rather than detailed insurance transitions), we assumed individuals who were insured prior to ACA did not become uninsured in 2014 to match overall insurance levels from BRFSS.

#### ACA medicaid expansion scenarios

In the Medicaid expansion scenarios, we utilized the same probabilities from the ACA scenario to determine whether an uninsured individual became newly insured in 2014. In addition, we identified uninsured individuals who fit eligibility guidelines for Medicaid under the expansion income and household size limits. We evaluated four hypothetical scenarios based on assumptions of enrollment among eligible individuals (low or high) and CRC screening compliance rates among newly insured individuals (low or high). We assessed enrollment rates in other states that expanded Medicaid and set the low and high rates to be comparable to the range observed (50% for low enrollment, 90% for high enrollment). We set low and high compliance rates to be the same as rates for individuals covered through Medicaid or private insurance, respectively (based on multilevel, multivariable statistical models). Thus, low and high compliance rates represent the most conservative and most generous estimates, respectively.

### Costs

We sought to estimate increased clinical care costs of screening and potential decreases in downstream costs of cancer treatment–a payer perspective, though we aggregate payers to focus on the overall balance of clinical costs. Costs for fecal testing ($6) and colonoscopy ($631) were based on Medicare reimbursement rates [33]. CRC treatment costs were included for the initial year of treatment [33]. In order to obtain a conservative estimate of cost savings, only short-term treatment costs were considered. All costs were adjusted to 2014 dollars using the medical component of the Consumer Price Index.

### Model runs and analysis

Given the large number of individuals simulated, we ran 5 replications of the model for each of the control and policy scenarios and compared the control scenario to each of the policy scenarios. We used the model to estimate the percent of African American and White males up-to-date with CRC screening, lifetime CRC cases prevented by stage, and lifetime CRC deaths prevented. We assessed the percent up-to-date with CRC screening in NC overall and by the Area Health Education Center’s (AHEC) regions. The regions were chosen because the AHEC agencies in each provide educational programs and services to improve health with a focus on underserved populations. We assessed the costs of CRC screening and the initial phase of treatment of the cohort of African American and White males. Costs were discounted using the conventional annual 3 percent rate [34].

## Results

**Fig 1:**
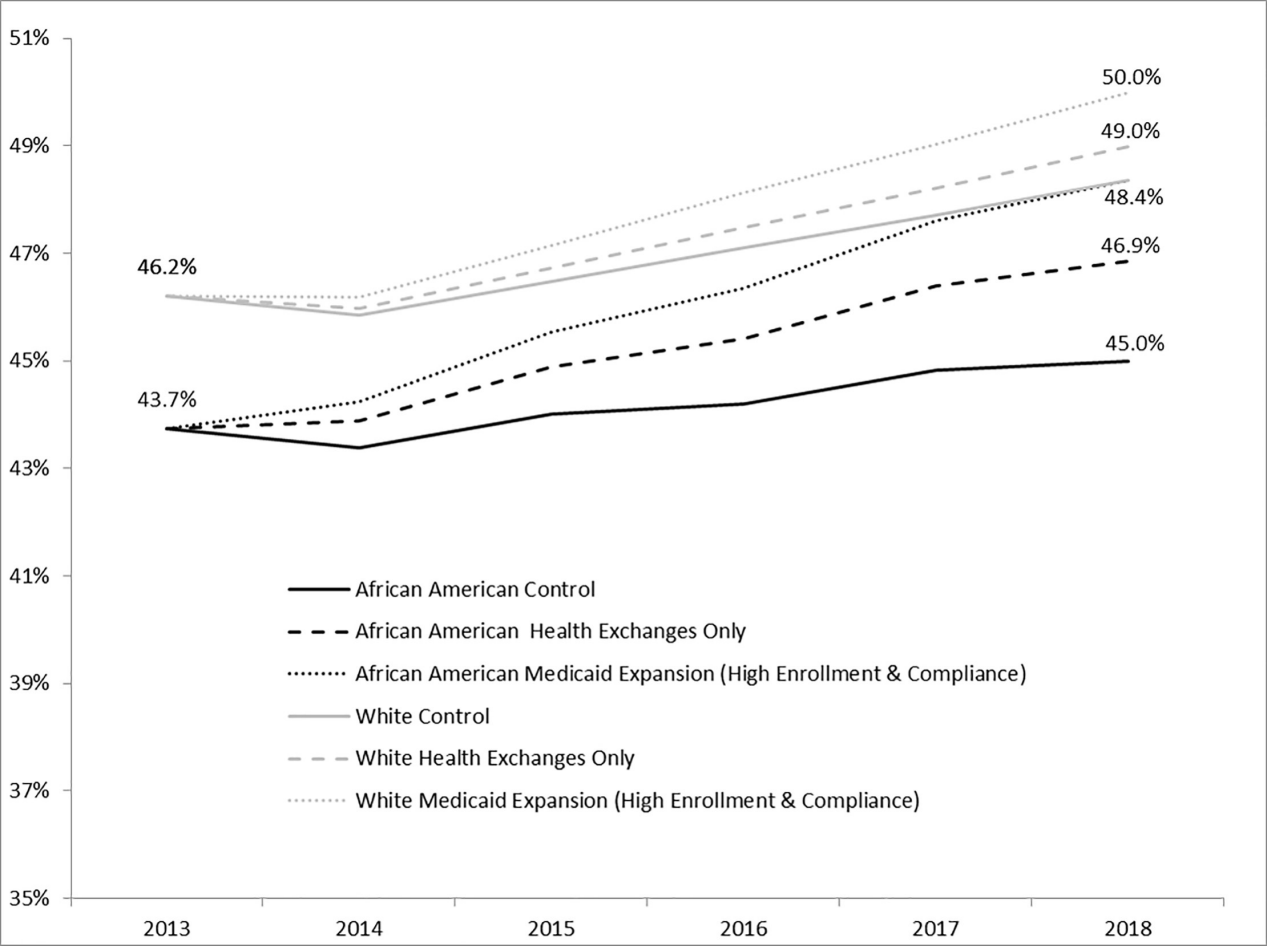
North Carolinian White and African American males predicted up-to-date with colorectal cancer screening by 2018 as a result of ACA policies.

The control and all ACA policy scenarios resulted in an increase over time (i.e., from 2013 onward) in the percent up-to-date with CRC screening among both White and African American males (Fig 1). By 2018, the control scenario indicated that 45.0% and 48.4% of age-eligible African American and White males, respectively, would be up-to-date with CRC screening. Compared to control, the ACA health exchange and Medicaid expansion policy scenarios all resulted in slightly larger increases in the percent up-to-date with CRC screening by 2018. The most generous ACA Medicaid expansion scenario (high enrollment and high compliance) resulted in 48.4% and 50.0% of African American and White age-eligible males, respectively, up-to-date with CRC screening by 2018. All other ACA Medicaid expansion scenarios also resulted in slight increases above the control scenario, but to a lesser degree.

**Fig 2:**
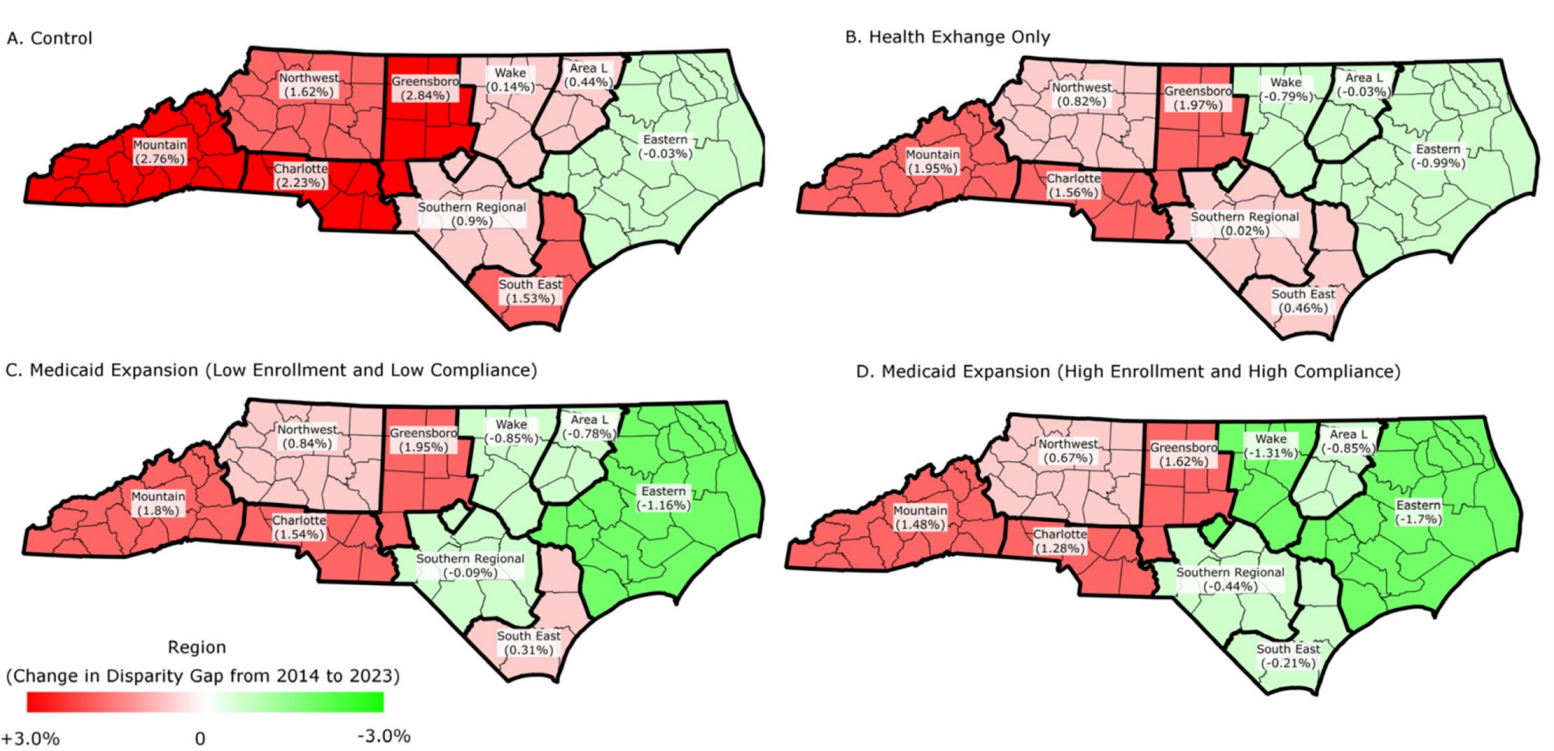
Change in disparity gap between White and African American males in the percent up-to-date with colorectal cancer screening from baseline to 2023 by NC geographic regions.

The ACA health exchange and Medicaid expansion policy scenarios had larger relative impacts on CRC screening rates for African American males than for White males and resulted in decreased disparities. In the control scenario, the disparity gap in CRC screening rates between African American and White males increased in all but one of nine major regions of NC (Fig 2). In contrast, the most generous Medicaid expansion scenario (high enrollment and high compliance) resulted in a decrease in the disparity gap in five regions, and the disparity gap increased to a lesser degree in the four other regions.

The ACA health exchange and Medicaid expansion scenarios prevented more CRC cases and deaths among both the African American and White male cohorts compared to the control scenario, but the impact was diminished in Medicaid expansion scenarios with low compliance. In all scenarios, the impacts of ACA health exchanges and Medicaid expansion were more substantial among African Americans than Whites (Table 1). Compared to the control scenario, the ACA health exchange scenario resulted in an average of 28.0 (standard deviation (SD) = 4.6) fewer CRC cases per 100,000 African American males and an average of 7.9 (SD = 1.0) fewer CRC cases per 100,000 White males. Compared to the control scenario, the ACA health exchange scenario resulted in an average of 16.0 (SD = 3.4) less deaths per 100,000 of African American males and 4.7 (SD = 1.0) fewer deaths per 100,000 of White males.

**Table 1:** CRC cases and deaths per 100,000 prevented by ACA’s health exchanges and Medicaid expansion among White and African American males in NC.

	Potential cases and deaths per 100,000	Potential cases and deaths prevented per 100,000	Potential cases and deaths per 100,000	Potential cases and deaths prevented per 100,000			
				Health Exchange + Medicaid expansion*			
	*Control (no ACA)*	*Health Exchange Only (Status Quo)*	*Health Exchange Only (Status Quo)*	*(High Enroll*, *Low Compliance)*	*(High Enroll*, *High Compliance)*	*(Low Enroll*, *Low Compliance)*	*(Low Enroll*, *High Compliance)*
		Mean (SD)		Mean (SD)			
***African American Males (N = 338*,*288)***							
**CRC Deaths**	**1,589.4**	16.0 (3.4)	**1,573.4**	6.6 (2.6)	14.7 (2.7)	3.4 (1.1)	12.7 (1.6)
**CRC Cases**	**3,178.7**	28.0 (4.6)	**3,150.6**	11.4 (3.0)	25.5 (5.6)	7.1 (4.6)	14.1 (3.8)
** Stage 1**	**727.6**	4.9 (1.8)	**722.8**	3.1 (0.9)	4.3 (2.5)	2.1 (1.4)	2.0 (1.4)
** Stage 2**	**842.8**	5.7 (3.1)	**837.2**	2.8 (0.9)	5.9 (2.8)	1.8 (0.8)	4.0 (2.3)
** Stage 3**	**728.4**	6.9 (1.8)	**721.6**	1.9 (1.1)	4.8 (2.0)	1.4 (0.8)	2.7 (1.2)
** Stage 4**	**879.8**	10.6 (2.6)	**869.1**	3.6 (2.7)	10.5 (4.5)	1.8 (1.4)	5.5 (2.2)
***White Males (N = 1*,*495*,*527)***							
**CRC Deaths**	**1,517.3**	4.7 (1.0)	**1,512.6**	4.7 (1.0)	9.5 (1.2)	2.3 (0.6)	5.1 (0.6)
**CRC Cases**	**3,132.9**	7.9 (1.0)	**3,125.0**	7.8 (2.0)	16.4 (5.0)	4.1 (0.9)	9.3 (0.3)
** Stage 1**	**724.0**	1.1 (0.5)	**722.9**	1.2 (0.5)	2.1 (0.3)	0.5 (0.4)	1.3 (0.2)
** Stage 2**	**821.3**	2.0 (0.6)	**819.4**	2.0 (0.6)	3.9 (1.0)	1.2 (0.3)	2.3 (0.4)
** Stage 3**	**754.2**	1.9 (0.7)	**752.2**	1.9 (0.8)	4.7 (0.8)	1.0 (0.5)	2.5 (0.7)
** Stage 4**	**833.4**	2.9 (0.4)	**830.5**	2.6 (0.6)	5.7 (0.4)	1.5 (0.5)	3.2 (0.4)

*assumptions about enrollment and compliance relative to Medicaid expansion only

**Fig 3:**
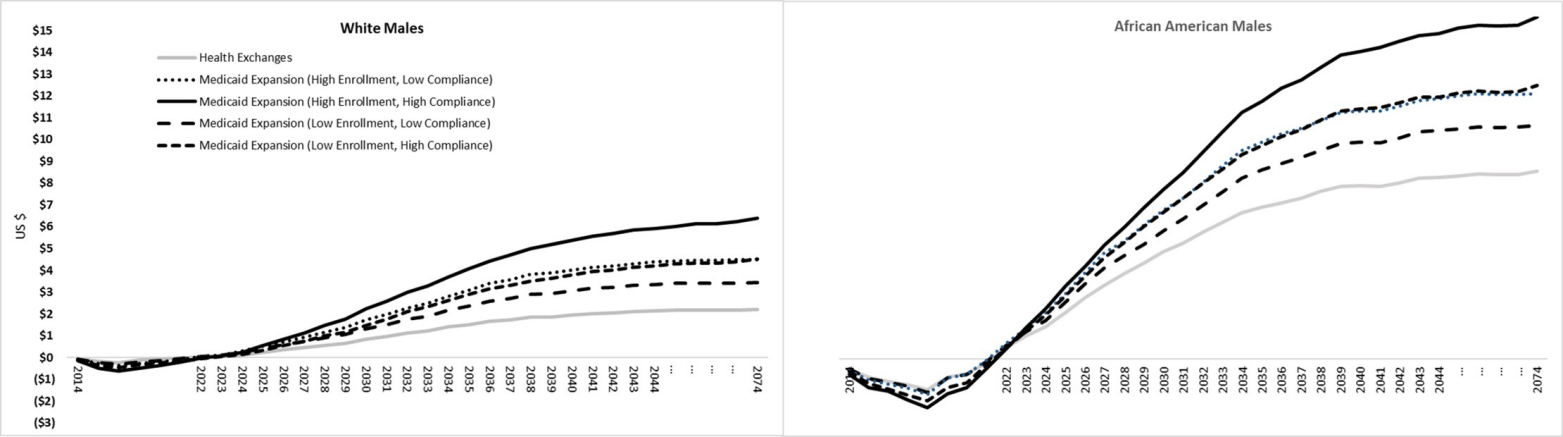
Differences in cumulative CRC screening and treatment cost savings per person between ACA policy scenarios and the control scenario among NC White and African American males.

The ACA health exchange and Medicaid expansion policy scenarios all incurred higher initial costs compared to the control scenario, but all policy scenarios were cost-saving in the long-term for both African American and White males (Fig 3). Based on our model, the ACA health exchange and Medicaid expansion scenarios were costlier for approximately 8 or 9 years (until 2022 or 2023) and became cost-saving in subsequent years when savings in cancer treatment overtook increased costs of screening. Initial costs were lowest for the ACA health exchange scenario compared to the Medicaid expansion scenarios, but Medicaid expansion resulted in greater cumulative cost savings in all scenarios. Relative to the control scenario, the ACA health exchange scenario resulted in $3.3 million cumulative (over a 60-year period) cost savings for White males and $2.8 million for African American males. The Medicaid expansion (high enrollment and high compliance) scenario resulted in $9.6 million in cumulative cost savings for White males and $5.1 million for African American males. In all scenarios, most cost-savings were realized within 30 years (by 2044).

The ACA policy scenarios resulted in higher per person costs for African American than White males in the short term, but the cumulative cost savings per person were also substantially higher for African Americans over the long-term (Fig 3). For example, in the most generous Medicaid expansion scenario (high enrollment and compliance), the costs among African American males were highest in 2018 at $2.13 per person, but subsequent savings in CRC screening and treatment costs led to a cumulative cost saving of $15.01 per African American male. Compared to African American males, the same scenario resulted in less initial costs per White male ($0.63 per person in 2018), but also resulted in less cumulative cost savings ($6.40 per person).

## Discussion

Our findings show that increased insurance coverage due to ACA health exchanges is likely to improve CRC screening and reduce CRC incidence and death over time with substantial cost-savings, highlighting the significant need for policies that stabilize health insurance markets. Medicaid expansion may also hold untapped potential to further reduce persistent CRC disparities in non-expansion states. Using NC as a case example, our simulation model predicted increases in the percent-up-to-date with CRC screening due to Medicaid expansion among African Americans who are disproportionately at risk for CRC morbidity and mortality. Population-level impact of expanding Medicaid eligibility in NC could result in hundreds of CRC deaths averted among non-Hispanic White and African American men. Such state-level achievements in CRC screening uptake would help to advance our nation’s progress towards the National CRC Roundtable’s goals of increasing CRC screening to 80% and preventing new CRC cases and deaths in every community [35]. Our results provide a compelling example of the positive impacts on CRC screening uptake and deaths that other non-expansion states might observe as a consequence of Medicaid expansion.

Many holdout states attribute their decision to forgo Medicaid expansion because of concerns over long-term costs and sustained federal commitment to Medicaid cost-sharing [36]. Yet, we detected potential long-term cost savings for CRC screening and treatment for African American men, which could offset excess medical expenditures and have a positive impact on the national economy. Admittedly, the federal matching assistance percentage (FMAP) was designed to phase-down from 100% to 90% [37]. However, competing evidence indicates that while states might experience an initial increase in costs associated with Medicaid expansion, these are projected to flatten and should eventually result in significant cost savings and revenue gains downstream [38]. Our findings support this supposition. Holdout states also express concerns over the influx of new Medicaid enrollees and the potential systems-level and provider burdens their enrollment may produce. While costs associated with Medicaid expansion are being debated, many low-income, uninsured individuals outside of current Medicaid eligibility requirements in holdout states found themselves choosing between paying higher marketplace premiums or the tax penalties imposed by the individual mandate, which was removed beginning in 2019. Many chose the latter meaning they will be even less likely to obtain timely and life-saving screenings for preventable conditions like CRC.

We concede that eliminating CRC disparities will require broader attention to the social determinants of health and personal health behaviors that help catalyze them. While we recognize that reductions in CRC incidence and mortality will not be fully achieved by expanding health insurance access, our findings suggest Medicaid expansion represents a “low hanging fruit” policy opportunity to reduce disparities. We are especially encouraged that even in the most conservative policy scenarios, CRC screening rates improved among all men and disparities were reduced. Even more encouraging was the finding suggesting that policy interventions like Medicaid expansion might also save American lives–an unquantifiable population health gain.

There are important limitations to consider when interpreting results from this study. Although the model differentiated rates of adenoma incidence by race and age, it assumes that the anatomic distribution and progression of CRC is the same between African Americans and Whites. There are studies that indicate there may be differences in genetic susceptibility, anatomic locations, and aggressiveness of tumors between African Americans and Whites [39–41]. Yet, the evidence remains mixed and it is uncertain the extent and direction to which these differences would change our results. Due to challenges inherent in our currently available data sources, we were unable to directly estimate the impact of obtaining insurance through health insurance exchanges on CRC screening in North Carolina. For example, with insurance claims data, the challenge is that data on screening only becomes available after screening (pre-insured screening rates are difficult to track). Thus, we made the best use of currently available data. However, as more research and data becomes available, analyses should be updated and integrated into modeling efforts.

Given our intent to assess the balance of clinical care costs overall, the model did not attribute costs by payer and makes simplifying cost assumptions. The study does not provide information on the distribution of costs between private and public funders. We did not include costs of administering ACA or Medicaid expansion, as the effects of each extend far beyond the focus of this analysis (CRC outcomes). However, including some component of administration costs would diminish the potential cost savings. To be conservative and focus on more proximal costs, we only included the initial phase (12 months) of treatment costs following a CRC diagnosis (and did not include the costs of continuing surveillance and end-of-life care). Including the initial phase only renders cost savings estimates lower than they are actually likely to be. Finally, evidence is only now emerging about enrollment in new healthcare coverage options due to ACA and adherence to CRC screening, and we had to make assumptions about the future uptake of insurance and adherence to screening in NC over time. We attempted to provide scenarios of Medicaid expansion that provide a plausible range of potential outcomes.

## Conclusions

The ACA and its provisions for Medicaid expansion resulted in increased insurance coverage for lower income men–a group traditionally ineligible for Medicaid. Medicaid expansion in holdout states like NC has the potential to reduce racial CRC disparities among men, which will likely worsen in the absence of policy interventions. Our investigation advances our understanding of the prospective, long-term benefits of ACA and Medicaid expansion in reducing preventable CRC morbidity and mortality. Our results suggest that leveraging opportunities created by Medicaid expansion to increase access to early CRC detection and screening in one state may save hundreds of lives. The national impact of ACA’s health exchanges and Medicaid expansion on CRC mortality is likely much larger. Future studies should replicate similar simulation research in other states and estimate the potential impact of various proposed healthcare reform policies on additional health outcomes. The need for such evidence is critical to state and national policymakers as they contemplate newly proposed legislation that will drastically change health insurance markets and Medicaid coverage and alter our nation’s capacity to reduce health disparities in populations vital to our capacity to compete in the global marketplace.
